# Early life gut microbiota is associated with rapid infant growth in Hispanics from Southern California

**DOI:** 10.1080/19490976.2021.1961203

**Published:** 2021-08-23

**Authors:** Tanya L. Alderete, Roshonda B. Jones, Justin P. Shaffer, Elizabeth A. Holzhausen, William B. Patterson, Elham Kazemian, Lida Chatzi, Rob Knight, Jasmine F. Plows, Paige K. Berger, Michael I. Goran

**Affiliations:** a Department of Integrative Physiology, University of Colorado Boulder, Boulder, CO, USA; b Department of Pediatrics, The Saban Research Institute, Children's Hospital Los Angeles, Los Angeles, CA, USA; c Department of Pediatrics, University of California San Diego, La Jolla, CA, USA; d Department of Population and Public Health Sciences, Keck School of Medicine, University of Southern California, Los Angeles, CA, USA; e Center for Microbiome Innovation, University of California San Diego, La Jolla, CA, USA; f Department of Computer Science & Engineering, University of California San Diego, La Jolla, CA, USA; g Department of Bioengineering, University of California San Diego, La Jolla, CA, USA

**Keywords:** Infant growth, gut microbiota, hispanics, obesity, growth trajectories

## Abstract

We aimed to determine if the newborn gut microbiota is an underlying determinant of early life growth trajectories. 132 Hispanic infants were recruited at 1-month postpartum. The infant gut microbiome was characterized using 16S rRNA amplicon sequencing. Rapid infant growth was defined as a weight-for-age z-score (WAZ) change greater than 0.67 between birth and 12-months of age. Measures of infant growth included change in WAZ, weight-for-length z-score (WLZ), and body mass index (BMI) z-scores from birth to 12-months and infant anthropometrics at 12-months (weight, skinfold thickness). Of the 132 infants, 40% had rapid growth in the first year of life. Multiple metrics of alpha-diversity predicted rapid infant growth, including a higher Shannon diversity (OR = 1.83; 95% CI: 1.07–3.29; *p* = .03), Faith’s phylogenic diversity (OR = 1.41, 95% CI: 1.05–1.94; *p* = .03), and richness (OR = 1.04, 95% CI: 1.01–1.08; *p* = .02). Many of these alpha-diversity metrics were also positively associated with increases in WAZ, WLZ, and BMI z-scores from birth to 12-months (p_all_<0.05). Importantly, we identified subsets of microbial consortia whose abundance were correlated with these same measures of infant growth. We also found that rapid growers were enriched in multiple taxa belonging to genera such as *Acinetobacter, Collinsella, Enterococcus, Neisseria*, and *Parabacteroides*. Moreover, measures of the newborn gut microbiota explained up to an additional 5% of the variance in rapid growth beyond known clinical predictors (R^2^ = 0.37 vs. 0.32, *p* < .01). These findings indicate that a more mature gut microbiota, characterized by increased alpha-diversity, at as early as 1-month of age, may influence infant growth trajectories in the first year of life.

## Introduction

A prominent concern with the obesity epidemic is the high rate of obesity present among children^1,2^ which is especially evident among Hispanics, a disparity that is manifested in early life.^1,3^ Childhood obesity may persist into adulthood, thereby contributing to widespread cardiometabolic comorbidities.^4–6^ Beyond maternal obesity,^7,8^ socioeconomic status,^9,10^ and early life nutrition,^7,11–13^ emerging evidence indicates that the infant gut microbiota may influence growth trajectories in the first year of life,^14–17^ which may in turn predict future obesity.^3,18,19^ Given that rapid infant growth is a potential determinant of overweight/obesity in school-age children,^20^ the developing gut microbiota may be an important predictor for rapid infant growth and childhood obesity.

Previous studies have shown that the composition of the infant gut microbiota is associated with measures of infant growth and childhood obesity risk.^14,21–26^ For instance, a greater proportion of *Staphylococcus aureus*^25^ and elevated richness of Firmicutes^26^ in early infancy have been correlated with the development of overweight and obesity during childhood. To our knowledge, no previous studies have assessed the impact of the composition of the infant gut microbiota on detailed measures of infant growth among Hispanic infants, a group with high rates of infant onset obesity.^2^ Therefore, the primary objective of this study was to determine whether the infant gut microbiota at 1-month of age was associated with rapid growth from birth to 12-months of age in a subset of mother-infant dyads from the Southern California Mother’s Milk Study. We hypothesized that greater maturation of the newborn gut microbiota, characterized by increased alpha-diversity and beta-diversity,^27^ would predict more rapid infant growth. As a secondary aim, we investigated whether gut bacterial diversity and/or specific gut microbial taxa were associated with additional measures of infant growth (i.e., weight-for-age z-score [WAZ], weight-for-length z-score [WLZ], body mass index [BMI] z-score, anthropometrics).

## Results

### General characteristics

As shown in Table 1, mothers were evenly split by pre-pregnancy BMI status where 37 (28%) were normal weight, 50 (38%) were overweight, and 45 (34%) were obese. Overall, 54 (40%) infants exhibited rapid growth from birth to 12-months of age. Infants with rapid growth had a significantly lower birth weight (*p* < .001) and length (*p* < .001) compared to those without rapid growth. Among maternal and infant characteristics, only infant birth weight (OR = 0.03, 95% CI: 0.006–0.10; *p* < .001) and length (OR = 0.76; 95% CI, 0.63–0.89; *p* = .001), infant weight (OR = 0.36; 95% CI: 0.16–0.75; *p* = .008) and length at 1-month (OR = 0.74; 95%, CI: 0.59–0.90; *p* = .004) were associated with rapid infant growth. For example, there were no differences in maternal characteristics between rapid vs. non-rapid growers, including maternal age at birth, pre-pregnancy BMI, mode of delivery (vaginal vs. cesarian), socioeconomic status, or maternal diet at 1-month postpartum (Table 1: p_all_ ≥0.40).

**Table 1.** Characteristics of mother-infant dyads from the Southern California mother’s milk study at 1-month of infant age

	Overall (n = 132)	Rapid Growth (n = 54)	Non-Rapid Growth (n = 78)	Crude OR (95% CI)	Crude OR *p*-value
***Maternal Characteristics***					
Age at Infant Birth (years)	29.5 ± 6.3	29.3 ± 6.2	29.7 ± 6.4	0.99 (0.94,1.0)	0.72
Pre-Pregnancy BMI (kg/m^2^)	28.4 ± 5.7	28.7 ± 5.4	28.1 ± 6.0	1.0 (0.96, 1.1)	0.51
Delivery (vaginal^a^, cesarian, %vaginal)	98, 34, 74.2%	38, 16, 70.4%	60, 18, 76.9%	1.4 (0.64, 3.1)	0.40
SES Index^b^	26.9 ± 12.2	26.2 ± 11.7	27.3 ± 12.6	0.99 (0.96, 1.0)	0.61
Pre-Pregnancy Weight Status					
Normal Weight	37, 28.0%	12, 22.2%	25, 32.1%	Ref	Ref
Overweight	50, 37.9%	22, 40.7%	28, 35.9%	1.6 (0.68, 4.0)	0.28
Obese	45, 34.1%	20, 37.0%	25, 32.1%	1.7 (0.67, 4.2)	0.27
Weight Status at 1-Month Postpartum					
Normal Weight	23, 17.4%	7, 13.0%	16, 20.5%	Ref	Ref
Overweight	48, 36.4%	20, 37.0%	28, 35.9%	1.6 (0.58, 4.9)	0.36
Obese	61, 46.2%	27, 50.0%	34, 43.6%	1.8 (0.67, 5.3)	0.25
***Maternal Dietary Measures at 1-Month***					
Total energy intake (kcals)	1724.9 ± 534.73	1682.7 ± 404.06	1761.8 ± 609.9	1.00 (1.00, 1.00)	0.45
Fat intake (g)	58.55 ± 23.09	56.918 ± 19.272	59.687 ± 25.462	0.99 (0.98, 1.01)	0.50
Protein intake (g)	76.487 ± 23.305	74.555 ± 17.79	77.825 ± 26.288	0.99 (0.98, 1.01)	0.42
Carbohydrate intake (g)	229.5 ± 81.701	223.17 ± 66.787	233.89 ± 91.299	1.00 (0.99, 1.00)	0.45
***Infant Characteristics***					
Age (days)	32.8 ± 5.2	33.4 ± 5.3	32.4 ± 5.2	1.0 (0.97, 1.1)	0.28
Infant Sex (female^a^, male, %female)	72, 60, 54.5%	30, 24, 55.6%	42, 36, 53.8%	0.93 (0.46, 1.9)	0.84
Delivery					
On-Time (n, %)^a^	64, 48.5%	26, 48.1%	38, 48.7%*	Ref	Ref
Late (n, %)	36, 27.3%	9, 16.7%	27, 34.6%	0.49 (0.19, 1.2)	0.12
Early (n, %)	32, 24.2%	19, 35.2%	13, 16.7%	2.1 (0.91, 5.2)	0.08
Birth Weight (kg)	3.4 ± 0.42	3.1 ± 0.36	3.6 ± 0.37***	0.03 (0.006, 0.10)	**<0.001**
Birth Length (cm)	50.5 ± 2.4	49.7 ± 2.3	51.1 ± 2.3***	0.76 (0.63, 0.89)	**0.001**
Weight (kg)	4.6 ± 0.49	4.5 ± 0.48	4.7 ± 0.48**	0.36 (0.16, 0.75)	**0.008**
Length (cm)	54.2 ± 1.8	53.6 ± 1.8	54.6 ± 1.8**	0.74 (0.59, 0.90)	**0.004**
Weight for Length z-Score	0.65 ± 1.3	0.70 ± 1.4	0.62 ± 1.3	1.0 (0.81, 1.4)	0.73
Body Mass Index z-Score	0.59 ± 1.1	0.47 ± 1.1	0.67 ± 1.1	0.84 (0.60, 1.2)	0.29
Antibiotics at 1-month (no^a^/yes, %yes)^c^	116, 15, 11.5%	47, 7, 13.0%	69, 8, 10.4%	1.28 (0.42, 3.81)	0.65
***Infant Dietary Measures***					
Breast Feedings/Day (≥8^a^, <8, % ≥8)	97, 35, 73.5%	37, 17, 68.5%	60, 18, 76.9%	1.5 (0.70, 3.4)	0.28
Formula Feeding/Day (yes/no^a^)	51, 81, 38.6%	18, 36, 33.3%	33, 45, 42.3%	1.5 (0.72, 3.1)	0.30
Age of Solid Food Introduction (months)	5.8 ± 1.5	5.7 ± 1.3	5.8 ± 1.6	0.93 (0.72, 1.2)	0.56

Table 1. Baseline (1-month) characteristics of 132 Hispanic mother-infant dyads from the Southern California Mother’s Milk Study. Pre-pregnancy BMI, infant sex, birth weight and birth length are also shown. Data are reported mean and standard deviation (SD) unless otherwise noted. Logistic regression was used to determine the crude odds ratio (OR) and 95% confidence intervals (CIs) for each baseline variable and rapid infant growth from birth to 12-months of age where the ^a^reference group is indicated. Total samples sizes include ^b^n = 130 for the Hollingshead Four Factor Index as a measure of socioeconomic status (SES) (rapid n = 53 and non-rapid n = 77) and ^c^n = 131 for infant antibiotic exposure in the first month of life. For continuous variables, independent parametric or non-parametric t-tests were used to test for differences between infants with rapid and non-rapid growth. For categorical variables, chi-square tests were used to test for differences between infants with rapid and non-rapid growth. Statistical significance between rapid and non-rapid growers corresponds to ***p < 0.001, **p < 0.01, and *p < 0.05.

### Gut bacterial alpha-diversity was associated with rapid growth

**Figure 1.**
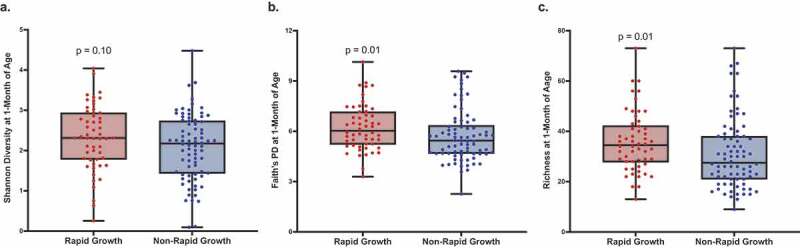
Gut bacterial alpha-diversity was higher in rapid compared to non-rapid growers at 1-month of age

While there were no observed differences in measures of beta-diversity, we observed significant differences in measures of alpha-diversity by infant growth status. Specifically, Faith’s PD (*p* = .01) and sOTU richness (*p* = .01) were higher among rapid- compared to non-rapid growers (Figure 1) where a higher Faith’s PD (OR = 1.41; 95%, CI: 1.05–1.94; *p* = .03) and richness (OR = 1.04; 95%, CI: 1.01–1.08; *p* = .02) also predicted rapid infant growth (Table 2). Greater maturation of the infant gut microbiota was associated with rapid infant growth after adjusting for birth weight and birth length. For example, infant gut microbial Shannon diversity at 1-month of age was associated with rapid infant growth from birth to 12-months (OR = 1.83; 95% CI, 1.07–3.29, *p* = .03). We performed several sensitivity analyses, adjusting for maternal BMI, infant exposure to antibiotics, and infant diet at 1-month of age and found the results largely unchanged by inclusion of these additional covariates. Results of these analyses suggest that these maternal and infant characteristics do not confound the present results and are included in Supplemental Tables 1–8. Lastly, we were interested in determining if infant gut bacterial diversity improved our ability to predict rapid infant growth when compared to known clinical predictors, such as maternal age, pre-pregnancy BMI, delivery mode, measure of socioeconomic status (SES), infant age and sex, birth weight and length, breast feedings per day, formula consumption, age of solid food introduction, and time of delivery. Including Shannon diversity, Faith’s PD, or richness at 1-month explained an additional 3% (R^2^ = 0.32 vs. 0.29, *p* = .06), 2% (R^2^ = 0.31 vs. 0.29, *p* = .14), and 2% (R^2^ = 0.31 vs. 0.29, *p* = .09) of the variance in rapid infant growth compared to known clinical predictors, respectively.

**Table 2.** Greater gut bacterial alpha-diversity were associated with rapid infant growth from birth to 12-months of age

	Adjusted OR (95% CI)	*p*-value
Alpha-Diversity Indices		
Shannon diversity	1.83 (1.07, 3.29)	**0.03**
Faith’s PD	1.41 (1.05, 1.94)	**0.03**
Richness	1.04 (1.01, 1.08)	**0.02**

Table 2. Multivariable odds ratios (OR) and 95% confidence intervals (CI) were calculated for these alpha-diversity indices, based on logistic regression with non-rapid growers as the referent, adjusting for birth length and birth weight. *P*-values in bold denote statistical significance for *p* < 0.05.

Figure 1. Boxplots for infant gut bacterial Shannon diversity, Faith’s PD, and sOTU richness at 1-month of age is shown for Hispanic infants with rapid (n = 54) and non-rapid growth (n = 78). *P*-values obtained from the Wilcoxon rank sum test with continuity correction.

### Gut bacterial alpha-diversity was associated with measures of growth

Shannon diversity, Faith’s phylogenetic diversity (PD), and richness of gut bacteria at 1-month were all associated with a greater increase in WAZ, WLZ, and BMI z-score in the first year of life after adjusting for birth weight and birth length (Table 3). As an example, Faith’s PD at 1-month was associated with a greater increase in infant WAZ (β = 0.14, CI: 0.01, 0.27; *p* = .03), WLZ (β = 0.14, CI: 0.02, 0.26; *p* = .03), and BMI z-score (β = 0.14, CI: 0.02, 0.27; *p* = .03) from birth to 12-months of age. Additionally, infant gut bacterial alpha-diversity metrics at 1-month were each associated with a greater infant weight and midthigh skinfold thickness at 12-months of age. Particularly, Shannon diversity (β = 0.27, CI: 0.05, 0.49; *p* = .02), Faith’s PD (β = 0.13, CI: 0.005, 0.25; *p* = .04), and richness (β = 0.01, CI: 0.0003, 0.03; *p* = .045) at 1-month were each associated with a greater infant weight. Shannon diversity (β = 1.36, CI: 0.41, 2.32; *p* = .006), Faith’s PD (β = 0.58, CI: 0.05, 1.12; *p* = .03), and richness (β = 0.07, CI: 0.01, 0.13; *p* = .02) at 1-month were also associated with a greater midthigh skinfold thickness at 12-months of age.

**Table 3.** Infant gut bacterial alpha-diversity was associated with measures of infant growth

	Shannon Diversity		Faith’s PD		Richness	
	Beta (95% CI)	*p*-value	Beta (95% CI)	*p*-value	Beta (95% CI)	*p*-value
***Growth Measures* *(Birth to 12-Months)***						
Weight-for-Age z-score	0.23 (−0.004, 0.47)	0.05	0.14 (0.01, 0.27)	**0.03**	0.01 (0.0003, 0.03)	**0.045**
Weight-for-Length z-score	0.21 (−0.01, 0.43)	0.06	0.14 (0.02, 0.26)	**0.03**	0.01 (0.002, 0.03)	**0.03**
BMI z-score	0.20 (−0.03, 0.42)	0.09	0.14 (0.02, 0.27)	**0.03**	0.02 (0.002, 0.03)	**0.03**
***Anthropometrics*** ***(12-Months)***						
Weight (kg)	0.27 (0.05, 0.49)	**0.02**	0.13 (0.005, 0.25)	**0.04**	0.01 (0.0003, 0.03)	**0.045**
Length (cm)	0.29 (−0.21, 0.80)	0.25	0.07 (−0.20, 0.35)	0.60	0.005 (−0.03, 0.04)	0.73
Tricep Skinfold Thickness (mm)	−0.13 (−0.64, 0.37)	0.60	0.07 (−0.21, 0.35)	0.62	0.01 (−0.02, 0.04)	0.53
Subscapular Skinfold Thickness (mm)	0.24 (−0.15, 0.64)	0.23	0.14 (−0.08, 0.36)	0.22	0.02 (−0.008, 0.04)	0.20
Suprailiac Skinfold Thickness (mm)	0.07 (−0.24, 0.37)	0.66	0.06 (−0.10, 0.23)	0.44	0.007 (−0.01, 0.03)	0.43
Midthigh Skinfold Thickness (mm)	1.36 (0.41, 2.32)	**0.006**	0.58 (0.05, 1.12)	**0.03**	0.07 (0.01, 0.13)	**0.02**

Table 3. Beta coefficients and 95% confidence intervals (CIs) from multivariable linear regression analysis used to examine the associations between measure of infant growth with measures of alpha-diversity. Models adjusted for birth weight and birth length. *P*-values in bold denote statistical significance for *p*-values <0.05.

### The infant gut microbiota at 1-month of age predicted rapid growth

**Figure 2.**
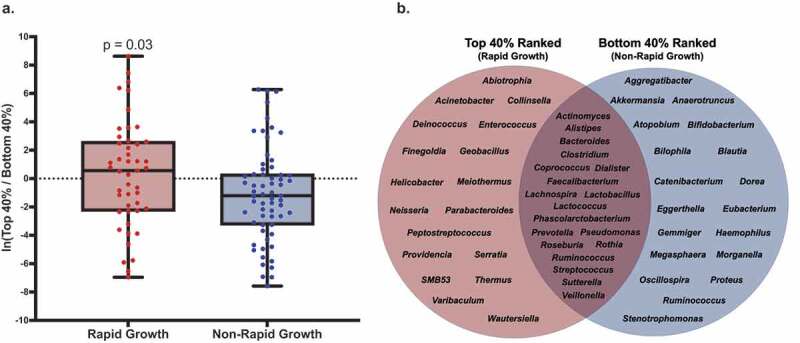
The Composition of the Infant Gut Microbiota at 1-Month of Age was Associated with Infant Growth in the First Year of Life

**Figure 3.**
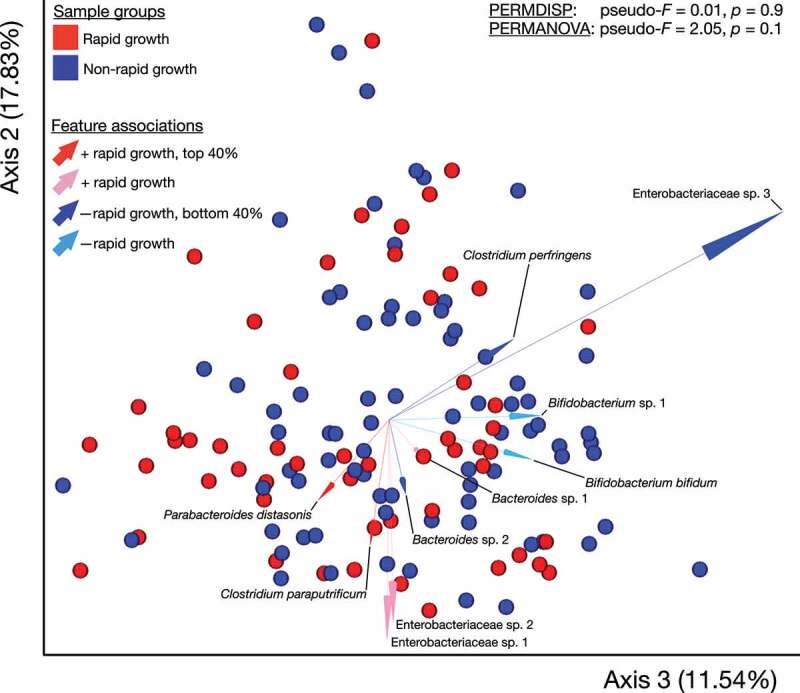
Robust aitchison principal coordinates analysis supports that infant gut microbial features are associated with rapid growth in the first year of life

Using a multinomial regression-based approach, we identified groups of gut bacteria that differed in abundance between rapid compared to non-rapid growers at 1-month of age (Figure 2a, *p* = .03). We examined these taxa at the genus-level and noted specific gut bacteria that were associated with rapid (n = 18) and non-rapid growers (n = 19) (Figure 2b). Taxa whose abundances were positively associated with rapid growers included the genera *Acinetobacter, Collinsella, Enterococcus, Neisseria, Parabacteroides*, and an unclassified genus belonging to the family *Ruminococcaceae*. Taxa whose abundances were negatively associated with rapid growers include the genera *Akkermansia, Bifidobacterium, Blautia, Eubacterium*, and *Ruminococcus*. In parallel, we identified support for several of these taxa being associated with rapid growth using an uninformed approach (Figure 3), including negative associations involving *Enterobacteriaceae* sp. 3 and *Clostridium perfringens*, and positive ones involving *Parabacteroides distasonis* and *Clostridium paraputriﬁcum*. Moreover, including the adjusted abundances of the top 40% of taxa whose abundances were identified to be positively associated with rapid growers (i.e., normalized to be compositionally-robust by taking the log-ratio of the abundances of those taxa with respect to the bottom 40%, and curated to exclude taxa not classified to at least the genus level and also genera present in bot top- and bottom groups) explained an additional 5% (R^2^ = 0.37 vs. 0.32, *p* < .01) of the variance in rapid infant growth compared to known clinical predictors, which included mother’s age, pre-pregnancy BMI, delivery mode, measure of SES, infant age and sex, birth weight and length, breast feedings per day, formula consumption, age of solid food introduction, and time of delivery. Next, we examined the abundances of the taxa identified as important above in a subset of infants with available sequencing data at 6-months of age (n = 92). These results indicate a tendency for the gut microbes identified at 1-month to remain important regarding distinguishing rapid growers at 6-months of age (Supplemental Figure 1).

Figure 2. (a) Comparison of the adjusted abundances of features associated with rapid- and non-rapid growth. The abundances were adjusted by taking the log-ratio of the top- and bottom 40% of features ranked based on their log-fold change with respect to rapid growth from our multinomial regression model. Selected features were additionally curated to exclude taxa not assigned to at least the genus level, and those assigned to genera present in both numerator (i.e., rapid growth) and denominator (i.e., non-rapid growth) groups. After curation, there were 25 and 27 features in numerator (i.e., rapid growth) and denominator (i.e., non-rapid growth) groups, respectively. Based on this selection, 110 of the 132 samples were included (83.3%). The multinomial regression model was additive, and adjusted for birth weight, birth length, and Faith’s PD. (b) The curated selection of the top- and bottom 40% of features (sOTU) grouped at the genus-level that were associated with infant growth. Taxa shared between groups are those excluded during curation in (A).

Figure 3. Robust Aitchison Principal Components Analysis (RPCA) biplot representing robust Aitchison distances between samples. Samples are colored based on rapid growth status. Arrows represent features, are colored by associations with rapid growth based on our multinomial regression model and annotated with their taxonomic classification (sOTUs lacking species-level classification were numbered within genera). Lengths of arrows correspond to correlations between feature loadings and the biplot axes. Samples near the arrow tips have strong correlations with respective features, and those near and beyond origins have negative correlations. Light-colored arrows indicate sOTUs with positive or negative associations with rapid growth, respectively, and dark arrows indicate sOTUs that were among the curated selection of the top- or bottom 40% of ranked associates, respectively. Note that axes 2 and 3 are represented (See Supplemental Figure 1 for axes 1 and 2). Results from permutational dispersion (PERMDISP) and multivariate analysis of variance (PERMANOVA) are shown.

**Figure 4.**
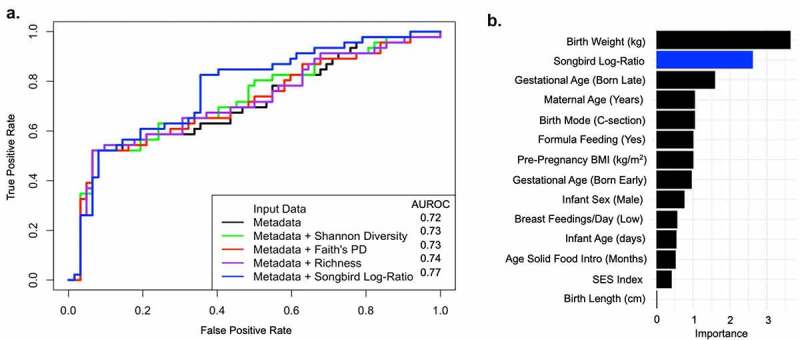
The relative importance of the newborn gut microbiota in predicting rapid infant growth compared to known clinical predictors

Our observation of increased ability to predict rapid infant growth using measures of the newborn gut microbiota is also supported by machine learning (Figure 4a), which shows that when predicting rapid vs. non-rapid growth, the discriminatory power of our random forest classifier (i.e., based on AUROC) increased to 0.77. This suggests that the newborn gut microbiota can be used to differentiate infants with rapid and non-rapid growth. Next, we examined the relative importance of the newborn gut microbiota compared to known clinical predictors for rapid infant growth. Based on variable importance (VIP) analysis we found that the adjusted abundances of the important taxa were ranked second (Figure 4b). Additionally, whereas infant birth weight had the highest VIP, other known clinical predictors were ranked lower than the adjusted abundances of important taxa, including mother’s age, pre-pregnancy BMI, delivery mode, SES, infant age and sex, birth length, breast feedings per day, formula consumption, age of solid food introduction, and time of delivery.

Figure 4. (a) The area under the receiver operating characteristic (AUROC) curve using leave-one-out predictions for rapid growth using a random forest classifier. The AUROC is indicated in the legend. The metadata correspond to known clinical predictors, including maternal age at birth (years), pre-pregnancy BMI (kg/m^2^), delivery mode (vaginal, c-section), measures of socioeconomic status (SES), infant age and sex, birth weight (kg) and length (cm), breast feedings per day (≥8, <8), formula feeding (yes/no), age of solid food introduction (days), and time of delivery (on-time, early [>2 weeks before due date], late [>2 weeks after due date]). (b) Importance based on variable importance (VIP) scores where a higher importance score indicates a greater contribution of each variable in predicting rapid infant growth. The Songbird log-ratio corresponds to the curated selection of the log-ratio of the top- and bottom 40% ranked sOTUs that were differentially associated with rapid infant growth in the first year of life.

### The infant gut microbiota at 1-month of age was associated with growth measures

The adjusted abundances of important taxa were significantly associated with additional measures of infant growth (Table 4). For example, the adjusted abundances of important taxa were associated with a greater increase in WAZ (β = 0.08, CI: 0.01, 0.15; *p* = .03) from birth to 12-months of age. Additionally, the composition of the newborn infant gut microbiota using these differential rankings was associated with a greater infant weight (β = 0.06, CI: 0.01, 0.12; *p* = .03) and skinfold thickness measures at 12-months. This included tricep (β = 0.13, CI: 0.0003, 0.26; *p* < .05), subscapular (β = 0.10, CI: 0.002, 0.21; *p* < .05), suprailiac (β = 0.10, CI: 0.02, 0.18; *p* = .02), and midthigh (β = 0.23, CI: −0.01, 0.47; *p* = .06) skinfold thickness measures. Among the subset of infants with available sequencing data at 6-months of age (n = 92), the adjusted abundances of taxa identified as important with respect to rapid growth 1-month of age were not associated with additional measures of infant growth (Supplemental Table 9).

**Table 4.** The newborn gut microbiota was associated with measures of infant growth

	Differentially Abundant Gut Microbes*	
	Beta (95% CI)	*p*-value
***Growth Measures*** ***(Birth to 12-Months)***		
Weight-for-Age z-score	0.08 (0.01, 0.15)	**0.03**
Weight-for-Length z-score	0.05 (−0.04, 0.14)	**0.28**
BMI z-score	0.06 (−0.02, 0.13)	**0.17**
***Anthropometrics*** ***(12-Months)***		
Weight (kg)	0.06 (0.01, 0.12)	**0.03**
Length (cm)	0.03 (−0.11, 0.17)	0.66
Tricep Skinfold Thickness (mm)	0.13 (0.0003, 0.26)	**<0.05**
Subscapular Skinfold Thickness (mm)	0.10 (0.002, 0.21)	**<0.05**
Suprailiac Skinfold Thickness (mm)	0.10 (0.02, 0.18)	**0.02**
Midthigh Skinfold Thickness (mm)	0.23 (−0.01, 0.47)	0.06

Table 4. Beta coefficients and 95% confidence intervals (CIs) from multivariable linear regression analysis used to examine the associations between measure of infant growth with gut microbial composition at 1-month of age. *Differentially abundant gut microbes are defined as a curated selection of the log-ratio of the top- and bottom 40% ranked sOTUs that were differentially associated with rapid infant growth in the first year of life. *P*-values in bold denote statistical significance for those <0.05.

## Discussion

We found that 40% of Hispanic infants from the Southern California Mother’s Milk Study exhibited rapid infant growth from birth to 12-months of age, which is a known risk factor for childhood obesity.^3,18,19^ Results from this study identified aspects of the newborn gut microbiota that distinguished rapid from non-rapid growers. Additionally, we found that higher gut microbial Shannon diversity and richness at 1-month predicted rapid infant growth from birth to 12-months of age. Further, we observed differences in the adjusted abundances of specific taxa in rapid compared to non-rapid growers, which represent 37 distinct bacterial genera. These differentially abundant taxa were also associated with greater increase in WAZ, WLZ, and BMI z-score from birth to 12-months of age. Lastly, gut bacterial Shannon diversity, richness, and the adjusted abundances of important taxa were associated with increased infant weight as well as greater midthigh skinfold thickness measures at 12-months. These results provide the first evidence that the newborn gut microbiota may contribute to rapid infant growth among Hispanics in the first year of life. Additionally, we found that the newborn gut microbiota was ranked second in importance when predicting rapid infant growth when compared to known clinical predictors such as maternal age, pre-pregnancy BMI, delivery mode, SES, infant age and sex, birth length, breast feedings per day, formula consumption, age of solid food introduction, and time of delivery.

There is growing evidence that the newborn gut microbiota is an underlying determinant of infant growth and childhood obesity^14,21–26^ through increased energy harvesting^28–30^ and/or production of gut bacterial derived metabolites that may contribute to obesity.^31–38^ As such, precocious maturation of the infant gut microbiota (e.g., increased diversity) may result in a more adult-like gut microbiome that is known to contribute to rapid growth.^39^ Increases in bacterial alpha-diversity may contribute to functional changes in the gut microbiome, including an increase in short-chain fatty acid production,^40^ genes responsible for complex carbohydrate- and starch metabolism,^41,42^ and an increase in intestinal energy extraction from food.^43^ Additionally, as infants develop, the gut microbial community becomes dominated by Bacteroidetes, Firmicutes, and Actinobacteria.^44^ Shifts to these dominant phyla include the addition of bacterial taxa involved in energy harvesting processes,^14,39,45^ which may impact infant growth trajectories and increase risk for childhood obesity. Thus, specific developmental processes related to gut bacterial composition and function may contribute to increased infant growth and childhood obesity.^46–48^

We found that measures of gut bacterial maturation at 1-month were associated with increased infant growth trajectories in the first year of life. To our knowledge, this is the first study to find that increased measures of alpha-diversity (e.g., Shannon, Faith’s PD, richness) were associated with greater increases in WAZ, WLZ, and BMI z-score from birth to 12-months of age as well as a greater infant weight and midthigh skinfold thickness at 12-months of age, suggesting that beyond growth trajectories, the gut microbiota may contribute to differences in infant body composition by 12-months of age. Our findings agree with most,^16,17,22^ but not all previous investigations.^49,50^ For example, higher 6-month Shannon diversity has been shown to be positively associated with changes in WAZ and WLZ from 6 to 12-months of age.^16^ Furthermore, higher gut bacterial Shannon diversity and richness from 3 to 4 months was associated with risk of overweight by 12-months of age.^17^ However, two studies have either failed to find differences in microbial diversity metrics between obese and normal weight Hispanic preschool children,^49^ or observed that gut bacterial diversity was inversely associated with BMI z-score among children 6–16 years of age.^50^ Inconsistency in the literature may be due to differences in the age groups being examined or the timepoint in which the gut microbiota was assessed.

This study utilized a compositionally-aware multinomial regression analysis^51^ to identify the top 40% of taxa whose abundances were positively associated with rapid infant growth in the first year of life. The adjusted abundances of these taxa were also associated with additional measures of infant growth, including greater increases in WAZ as well as a greater infant weight and skinfold thickness measures at 12-months of age. Next, we examined the 52 differentially ranked taxa at the genus level in order to gain a deeper insight into potentially important differences in gut bacterial composition among rapid compared to non-rapid growers. We found that rapid growers were already enriched at 1-month of age in multiple genera that have been linked with obesity (e.g., *Acinetobacter*,^15,52^ *Collinsella*,^53,54^ *Enterococcus*^55–57^). For example, two studies found an increased relative abundance of *Collinsella* among Mexican adolescents and children with obesity, respectively, compared to their normal weight peers.^53,54^ Additionally, studies examining Chinese children have observed a higher relative abundance of the *Enterococcus* genus among those with obesity.^56,57^ Non-rapid growers were enriched in genera linked with gut barrier integrity^58^ and immune maturation that may be protective against obesity in childhood (e.g., *Akkermansia*,^59^ *Bifidobacterium*,^60^ *Blautia*,^61^ *Eubacterium*^62^). For example, studies have found an inverse association between the relative abundance of *Blautia* and *Eubacterium* and levels of adiposity in children.^61,62^ Lastly, since these gut microbes were detected early in life, we explored whether the relative importance of the bacteria persisted beyond 1-month of life. Thus, we examined the group of gut microbiota identified as important for rapid growth at 1-month, in a subset of infants with available sequencing data at 6-months of age. Overall, we found a tendency for the adjusted abundances of gut microbes identified as important at 1-month to remain high in rapid- compared to non-rapid growers at 6-months of age. Whereas these results did not reach statistical significance (*p*-value = 0.2), these findings support the importance of the gut microbes identified at 1-month of age among a larger group of bacteria distinguishing rapid growers at six months. Additional members of the distinguishing group at 6-months that were not detected at 1-month of age may represent taxa present at 1-month that were not initially important, or they may represent those that were absent at 1-month but became established by 6-months of age. It is also possible that gut microbial profiles at 1-month of age predict the trajectory of the developing gut microbiome (composition and function), which may play a larger role in early life growth trajectories.

The strengths of the current study include early assessment of the newborn gut microbiota at 1-month of age and detailed longitudinal measures of infant growth trajectories among Hispanic infants that are at increased risk for childhood obesity. Additionally, this study included detailed information regarding potentially important confounding variables (e.g., mode of delivery, time of delivery, SES, pre-pregnancy BMI, maternal age, maternal and infant diet, birth weight and length). Despite these strengths, some potentially important variables were not included in the Mother’s Milk Study. For example, the current study lacked genetic information and paternal BMI which may impact infant growth. Whereas all participants in this study self-identified as being of Hispanic ethnicity, we did not directly assess country of origin, which may predict the composition of the gut microbiome.^63^ Some studies suggest that dietary intake during pregnancy may impact the maternal and infant gut microbiome.^64–67^ Since we did not have information regarding diet during pregnancy, we examined maternal macronutrient and energy intake at 1-month postpartum and did not observe any differences by infant growth status. The current study also did not examine human milk oligosaccharides that have been shown to impact the development of the infant gut microbiome.^68–71^ However, we found that the associations between the infant gut microbiota and rapid infant growth were largely unchanged after adjusting for infant diet (i.e., breast feedings per day and infant formula feedings per day). Additionally, infant diet was not associated with rapid infant growth in the first year of life. Cessation of breast feeding was not analyzed in this analysis; however, at 1-month of age none of the infants had ceased breast feeding. This study was also limited in that we could not identify specific gut bacterial species that may underlie the observed associations between the gut microbiota and increased infant growth trajectories. Lastly, the current study is limited in that it lacked repeated assessment of the infant gut microbiota throughout the first year of life;^72^ however, our results are consistent with previous studies that suggest that early developmental processes in the gut may contribute to childhood overweight and obesity.^14,21–26^

Our findings illustrate that the gut microbiota as early as 1-month of age predicts rapid infant growth and explains up to an additional 5% of the variance in rapid growth beyond known clinical predictors. These results suggest that the newborn gut microbiota may play an important role in the development of early life obesity in Hispanic children. Future work is needed to determine whether specific aspects of the infant gut microbiome (e.g., species, functional profiles) contribute to biological pathways known to be linked with obesity. Overall, findings from this study suggest that the gut microbiome should be examined as a potential target for childhood obesity prevention. For example, future studies should work to identify specific gut bacterial species that contribute to early life growth and development. Such studies may inform interventions aimed at modulating the gut microbiome at key developmental windows through the use of probiotics.

## Methods

### Participants

The study population was selected between 2016–2019 from the Southern California Mother’s Milk Study, which was designed to examine the association of breast milk factors on the infant gut microbiota and changes in infant growth among Hispanic mother-child dyads.^73,74^ At the time of this analysis, 135 infants had gut microbiota data at 1-month and 92 had gut microbiota data at 6-months. Infants who had microbiota data at 6-months did not differ substantially from those included in the 1-month sample (Supplemental Table 10). Study participants were recruited from clinics affiliated with the University of Southern California in Los Angeles County and other community clinics. The eligibility criteria included: healthy, term, singleton birth; self-identified Hispanic aged ≥18 years old at the time of delivery; 1-month postpartum; and intending to breastfeed for at least 3 months postpartum. Participants were excluded if: diagnosed with any medical condition which could affect metabolism, nutritional status, physical or mental health; taking medications that affect body weight/composition, insulin resistance, or lipid profiles; currently using tobacco or other recreational drugs; or diagnosed with fetal abnormalities. Written informed consent was obtained from all participants and the University of Southern California and Children’s Hospital Los Angeles Institutional Review Boards approved the study protocol.

### Study visits

Maternal weight was measured to the nearest 0.1 kg (Tanita, Model BC-549) and standing height was measured to the nearest 1 mm (Seca GmBH & Co. KG, Model Seca 126) to calculate BMI.^75^ Self-reported height and weight were used to assess pre-pregnancy BMI (kg/m^2^). Maternal diet was assessed using 24-hour dietary recalls at 1-month postpartum.^73,76^ Infant weight was measured in duplicate to the nearest 5 g by net difference of the mother with and without baby on a Tanita scale. Infant length was measured to the nearest 0.1 cm using an infantometer.^74^ Infant umbilical circumference was measured to the nearest 0.1 cm using a tape measure resistant to stretching.^74^ Infant skinfold thickness was measured using a caliper at the tricep, subscapular, suprailiac, and midthigh regions.^74^ Birth weight and length were obtained from hospital records. The measured average weight and length were then used to calculate WAZ, WLZ, and BMI z-scores using the WHO Child Growth Standards.^77^ Rapid infant growth was defined as a WAZ change >0.67 between birth and 12 months of age.^18,78,79^ Infant breast feedings per day (≥8, <8), formula feeding (yes/no), age of solid food introduction (months), and time of delivery (on-time, early [>2 weeks before due date], late [>2 weeks after due date]) were assessed using maternal questionnaires.^74^ Lastly, information regarding parental education and occupation was collected in order to calculate individual socioeconomic status (SES) based off of a modified version of the four-factor Hollingshead Index,^80^ where students, stay-at-home parents, and unemployed persons were assigned a score of zero in order to keep them in the analysis.^81^

### Gut microbiota

Infant stool samples were collected using OmniGene GUT kits at 1- and 6-months postpartum. DNA was extracted for 16S rRNA amplicon sequencing using the 515/806 barcoded primer pair (515 F: GTGCCAGCMGCCGCGGTAA, 806 R: GGACTACHVGGGTWTCTAAT) and then standardized in accordance with the Earth Microbiome Project. The 515/806 barcoded primer pair has been used for cross-cultural analysis that included infants.^82^ Paired-end, 2x150bp, next-generation sequencing was performed on the Illumina MiSeq platform available in the Institute for Genomic Medicine at the University of California San Diego.^83^ Demultiplexed files were processed using Qiita (https://qiita.ucsd.edu).^84^ Sequences were trimmed to a length of 150-bp, and examined using a reference-free method, Deblur,^85^ to remove suspected error sequences, provide amplicon sequence variants^86^ called sub-operational taxonomic units (sOTUs), and generate a feature-table with counts of each sOTU per sample. To generate a phylogeny, Deblur tag sequences were inserted into the GreenGenes 13_8 backbone phylogeny using SATÉ-enabled phylogenetic placement (SEPP),^87,88^ and all sOTUs not placed during SEPP were removed from the feature-table. sOTUs were assigned taxonomy^89^ using the GreenGenes 13_8 database trimmed to the V4 region and the q2-feature-classifier classify-sklearn (from the Quantitative Insights into Microbial Ecology 2 [QIIME2] tool).^90^

## Statistical analyses

### Gut bacterial community diversity

To normalize the sampling depth across samples, we rarefied the number of reads per sample to a read depth of 10,000, resulting in three samples being dropped. For alpha-diversity (within-sample diversity), we estimated sOTU richness, Shannon diversity, and Faith’s phylogenetic diversity (PD).^91,92^ Differences in alpha-diversity were examined using a Wilcoxon rank sum test with continuity correction. We estimated beta-diversity (between sample diversity) using weighted UniFrac, unweighted UniFrac, and the robust Aitchison distances from robust Aitchison principal components analysis (RPCA).^92^ We visualized the latter using RPCA, which is robust to sparsity (e.g., taxa absent in a majority of samples) and compositionality inherent in microbiome datasets. Lastly, we ran permutational dispersion analysis (PERMDISP) and permutational multivariate analysis of variance (PERMANOVA) on each distance matrix to examine the effect of infant growth status on bacterial community composition (n = 999 permutations per run).

### Descriptive statistics and infant growth analysis

Descriptive statistics (means and frequencies) of key variables were performed on the full sample and mean/frequency differences by growth status were assessed. Unadjusted logistic regression analysis was used to determine whether maternal and/or infant baseline characteristics predicted rapid infant growth. These results were used to identify potentially important covariates to include in our adjusted models, which included birth weight and length as well as 1-month weight and length. However, since birth weight and length were significantly correlated with 1-month measures (both r = 0.57 and *p* < .0001), we only examined birth weight and birth length. Finally, we performed several sensitivity analyses by adjusting for maternal and/or infant factors that are known to be associated with the developing gut microbiome (e.g., maternal BMI, infant antibiotic exposure, and infant diet). Adjusted logistic regression analyses were then performed to determine the associations between alpha-diversity metrics and rapid infant growth from birth to 12-months of age after adjusting for covariates (Table 1). Lastly, multivariable linear regression analyses were performed to determine if alpha-diversity was associated with measures of infant growth (e.g., WAZ, WLZ, anthropometric measures).

Songbird (v1.0.3)^51^ and Qurro (0.7.1)^93^ were used to calculate and display the differential ranks of sOTUs associated with infant growth status, respectively. Briefly, Songbird accounts for the compositional nature of microbiome data and uses a multinomial regression model to estimate differential rankings for features with respect to model variables. Models were adjusted for birth weight and birth length as well as Faith’s PD in order to examine whether sOTUs were associated with infant growth status independent of gut bacterial diversity. The full list of feature rankings from this analysis are included in Supplemental Table 11. Using Qurro, we then selected the top- and bottom 40% of ranked sOTUs, corresponding to those 40% of taxa whose changes in relative abundance across samples are most- or least-associated with rapid growth. Based on this selection, 110 of the 132 samples were included (83.3%). We curated this selection of sOTUs by excluding those lacking taxonomic classification to at least the genus level and also those genera that were present in both top- and bottom-ranked groups. A Wilcoxon rank-sum test was used to compare the log-ratio of the abundances of the curated selection of the top- and bottom ranked groups, between rapid- and non-rapid growers. We also examined the log-ratio of the same microbes for potential associations with measures of infant growth (e.g., WAZ, WLZ, anthropometric measures). Further, as a robustness check, we examined the sOTUs that were identified in the 1-month analysis at 6-months, for a subset of infants that had available sequencing data (n = 92). We found that there was a tendency for microbes identified at 1-month to persist at 6-months (see Supplemental Figure 2 and Supplemental Table 9).

Lastly, we determined whether assessment of the newborn gut microbiota could improve our ability to predict rapid infant growth beyond known clinical predictors using three approaches: 1) model fit based on McFadden pseudo R^2^ values and likelihood ratio tests, 2) microbiome-growth status associations using machine learning to in order to examine the area under the receiver operating characteristic (AUROC) curves using leave-one-out predictions for rapid growth with a random forest classifier, and 3) microbiome-growth status associations using interpretable machine learning variable importance (VIP) scores (*vip* package in R). Analyses were conducted using QIIME2 v.2020.11 and R (Version 3.6.2). Statistical significance was defined as a *p*-value<0.05 and 95% confidence intervals (CIs) are reported with effect estimates.

## Supplementary Material

Supplemental Material

## Concept and design

Alderete, Goran. **Acquisition, analysis, or interpretation of data**: All authors. **Drafting of manuscript**: Alderete, Jones, Shaffer, Patterson, Holzhausen, Kazemian. **Critical revision of the manuscript for important intellectual content**: All authors. **Statistical analysis**: Alderete, Jones, Shaffer, Holzhausen. **Supervision**: Alderete, Goran.

## Data sharing

Data available on request from the authors. The data that support the findings of this study are available from the corresponding author, MIG, upon reasonable request.
