# Supplementary material for: Early life gut microbiota is associated with rapid infant growth in Hispanics from Southern California
Source: Gut Microbes. 2021 Aug 23;13(1):1961203. doi: 10.1080/19490976.2021.1961203 (PMC8386720; doi:10.1080/19490976.2021.1961203)
Supplement: Supplemental Material [file KGMI_A_1961203_SM4506.zip › suppl/MM_Supplemental_Figures_Tables_02July21_Gut_Microbes.docx]

**Supplemental Figure 1: Robust Aitchison Principal Coordinates Analysis Supports that Infant Gut Microbial Features are Associated with Rapid Growth in the First Year of Life**


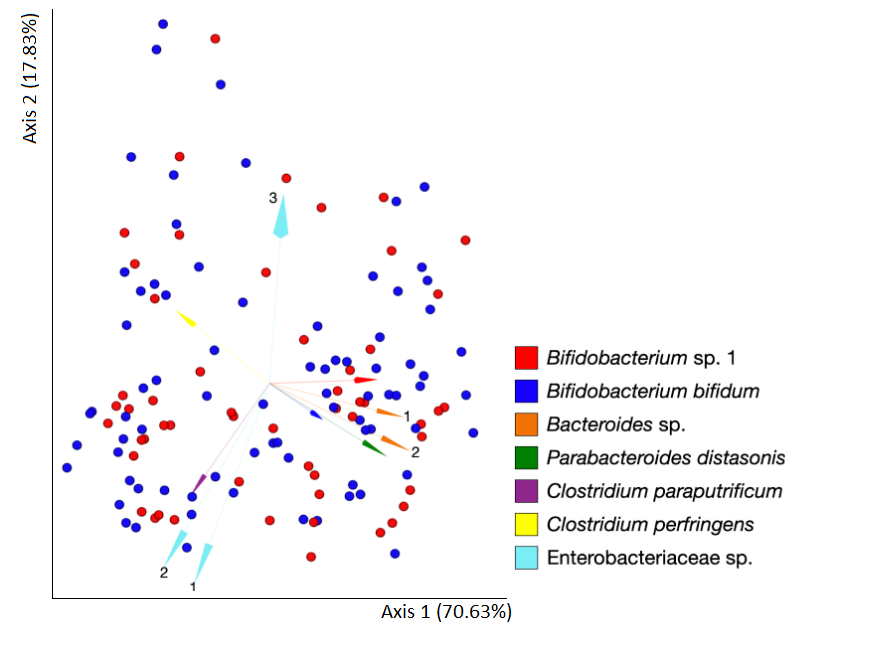


**Supplemental Figure 1**. Robust Aitchison Principal Components Analysis (RPCA) biplot representing robust Aitchison distances between samples. Samples are colored based on rapid growth. Arrows represent features and are colored by bacterial taxon with numbers indicating species for respective genera. Lengths of arrows correspond to correlations with feature loadings on respective axes. Samples near the arrow tips have strong correlations with respective features, and those near and beyond origins have negative correlations. This is the same plot as shown in **Figure 3**, showing axes 1 and 2 rather than 2 and 3.

**Supplemental Figure 2. The Composition of the Infant Gut Microbiota at 6-Months of Age was Associated with Infant Growth in the First Year of Life**

**Supplemental Figure 2**. The log-ratio of features associated with rapid- and non-rapid growth at 1-month in the 6-month gut microbiota samples (n = 92, p = 0.33).

**Supplemental Table 1. Greater Gut Bacterial Alpha-Diversity were Associated with Rapid Infant Growth from Birth to 12-Months of Age after Adjusting for Pre-Pregnancy BMI**

|  |  | **Adjusted OR**  **(95% CI)** | ***p*-value** |
| --- | --- | --- | --- |
| ***Alpha-Diversity Indices*** |  |  |  |
| Shannon diversity |  | 1.79 (1.04, 3.21) | **0.04** |
| Faith’s PD |  | 1.39 (1.03, 1.91) | **0.03** |
| Richness |  | 1.04 (1.01, 1.08) | **0.03** |

**Supplemental Table 1.** Multivariable odds ratios (OR) and 95% confidence intervals (CI) were calculated for these alpha-diversity indices, based on logistic regression with non-rapid growers as the referent, adjusting for birth length, birth weight, and maternal BMI. *P*-values in bold denote statistical significance for p<0.05.

**Supplemental Table 2**. **Infant Gut Bacterial Alpha-Diversity was Associated with Measures of Infant Growth after Adjusting for Pre-Pregnancy BMI**

|  | **Shannon Diversity** | | |  | **Faith’s PD** | |  | **Richness** | |
| --- | --- | --- | --- | --- | --- | --- | --- | --- | --- |
|  | **Beta**  **(95% CI)** |  | ***p*-value** |  | **Beta**  **(95% CI)** | ***p*-value** |  | **Beta**  **(95% CI)** | ***p*-value** |
| ***Growth Measures***  ***(Birth to 12-Months)*** |  |  |  |  |  |  |  |  |  |
| Weight-for-Age z-score | 0.22 (-0.014, 0.46) |  | 0.07 |  | 0.14 (0.010, 0.27) | **0.04** |  | 0.015 (0.00009, 0.029) | **0.049** |
| Weight-for-Length z-score | 0.20 (-0.017, 0.42) |  | 0.07 |  | 0.13 (0.014, 0.25) | **0.03** |  | 0.015 (0.0015, 0.028) | **0.03** |
| BMI z-score | 0.19 (-0.040, 0.42) |  | 0.1 |  | 0.14 (0.013, 0.27) | **0.03** |  | 0.016 (0.0019, 0.030) | **0.03** |
| ***Anthropometrics***  ***(12-Months)*** |  |  |  |  |  |  |  |  |  |
| Weight (kg) | 0.26 (0.039, 0.48) |  | **0.02** |  | 0.13 (0.003, 0.25) | **0.045** |  | 0.014 (0.00015, 0.027) | **0.048** |
| Length (cm) | 0.28 (-0.23, 0.78) |  | 0.28 |  | 0.070 (-0.21, 0.35) | 0.62 |  | 0.0049 (-0.026, 0.036) | 0.8 |
| Tricep Skinfold Thickness (mm) | -0.12 (-0.62, 0.39) |  | 0.64 |  | 0.075 (-0.20, 0.35) | 0.60 |  | 0.010 (-0.021, 0.041) | 0.5 |
| Subscapular Skinfold Thickness (mm) | 0.26 (-0.14, 0.65) |  | 0.20 |  | 0.14 (-0.08, 0.36) | 0.21 |  | 0.016 (-0.008, 0.040) | 0.2 |
| Suprailiac Skinfold Thickness (mm) | 0.067 (-0.24, 0.37) |  | 0.67 |  | 0.066 (-0.10, 0.23) | 0.44 |  | 0.0074 (-0.011, 0.026) | 0.4 |
| Midthigh Skinfold Thickness (mm) | 1.36 (0.40, 2.32) |  | **0.006** |  | 0.58 (0.044, 1.12) | **0.03** |  | 0.068 (0.0088, 0.13) | **0.03** |

**Supplemental Table 2.** Beta coefficients and 95% confidence intervals (CIs) from multivariable linear regression analysis used to examine the associations between measure of infant growth with measures of alpha-diversity. Models adjusted for birth weight, birth length, and maternal BMI. *P*-values in bold denote statistical significance for *p*-values <0.05.

**Supplemental Table 3. The Newborn Gut Microbiota was Associated with Measures of Infant Growth after Adjusting for Pre-Pregnancy BMI**

|  | **Differentially Abundant**  **Gut Microbes*** | | |  |
| --- | --- | --- | --- | --- |
|  | **Beta**  **(95% CI)** |  | ***p*-value** |  |
| ***Growth Measures***  ***(Birth to 12-Months)*** |  |  |  |  |
| Weight-for-Age z-score | 0.097 (0.024, 0.17) |  | **0.01** |  |
| Weight-for-Length z-score | 0.069 (-0.023, 0.16) |  | 0.14 |  |
| BMI z-score | 0.081 (0.0003, 0.16) |  | **0.049** |  |
| ***Anthropometrics***  ***(12-Months)*** |  |  |  |  |
| Weight (kg) | 0.12 (0.065, 0.18) |  | < **0.001** |  |
| Length (cm) | 0.037 (-0.10, 0.18) |  | 0.6 |  |
| Tricep Skinfold Thickness (mm) | 0.14 (0.008, 0.28) |  | **0.04** |  |
| Subscapular Skinfold Thickness (mm) | 0.08 (-0.026, 0.19) |  | 0.14 |  |
| Suprailiac Skinfold Thickness (mm) | 0.15 (0.071, 0.23) |  | **< 0.001** |  |
| Midthigh Skinfold Thickness (mm) | 0.37 (0.11, 0.63) |  | **0.005** |  |

**Supplemental Table 3.** Beta coefficients and 95% confidence intervals (CIs) from multivariable linear regression analysis used to examine the associations between measure of infant growth with gut microbial composition at 1-month of age. *Differentially abundant gut microbes are defined as a curated selection of the log-ratio of the top- and bottom 40% ranked sOTUs that were differentially associated with rapid infant growth in the first year of life. P-values in bold denote statistical significance for those <0.05.

**Supplemental Table 4. Greater Gut Bacterial Alpha-Diversity were Associated with Rapid Infant Growth from Birth to 12-Months of Age after Adjusting for Infant Antibiotic Exposure in the First Month of Life**

|  |  | **Adjusted OR**  **(95% CI)** | ***p*-value** |
| --- | --- | --- | --- |
| ***Alpha-Diversity Indices*** |  |  |  |
| Shannon diversity |  | 2.05 (1.17, 3.78) | **0.02** |
| Faith’s PD |  | 1.43 (1.06, 1.98) | **0.02** |
| Richness |  | 1.04 (1.01, 1.08) | **0.02** |

**Supplemental Table 4.** Multivariable odds ratios (OR) and 95% confidence intervals (CI) were calculated for these alpha-diversity indices, based on logistic regression with non-rapid growers as the referent, adjusting for birth length, birth weight, and antibiotic use during the first month of life. *P*-values in bold denote statistical significance for p<0.05.

**Supplemental Table 5**. **Infant Gut Bacterial Alpha-Diversity was Associated with Measures of Infant Growth after Adjusting for Infant Antibiotic Exposure in the First Month of Life**

|  | **Shannon Diversity** | | |  | **Faith’s PD** | |  | **Richness** | |
| --- | --- | --- | --- | --- | --- | --- | --- | --- | --- |
|  | **Beta**  **(95% CI)** |  | ***p*-value** |  | **Beta**  **(95% CI)** | ***p*-value** |  | **Beta**  **(95% CI)** | ***p*-value** |
| ***Growth Measures***  ***(Birth to 12-Months)*** |  |  |  |  |  |  |  |  |  |
| Weight-for-Age z-score | 0.19 (-0.09, 0.47) |  | 0.18 |  | 0.12 (-0.03, 0.27) | 0.13 |  | 0.01 (-0.005, 0.29) | 0.17 |
| Weight-for-Length z-score | 0.24 (-0.11, 0.58) |  | 0.17 |  | 0.24 (0.06, 0.42) | **0.01** |  | 0.02 (0.004, 0.04) | **0.02** |
| BMI z-score | 0.18 (-0.12, 0.49) |  | 0.24 |  | 0.19 (0.02, 0.35) | **0.02** |  | 0.02 (0.0015, 0.038) | **0.03** |
| ***Anthropometrics***  ***(12-Months)*** |  |  |  |  |  |  |  |  |  |
| Weight (kg) | 0.32 (0.10, 0.55) |  | **0.006** |  | 0.14 (0.01, 0.26) | **0.03** |  | 0.015 (0.001, 0.03) | **0.04** |
| Length (cm) | 0.38 (-0.15, 0.91) |  | 0.16 |  | 0.10 (-0.19, 0.39) | 0.50 |  | 0.008 (-0.02, 0.04) | 0.62 |
| Tricep Skinfold Thickness (mm) | -0.10 (-0.61, 0.41) |  | 0.71 |  | 0.06 (-0.21, 0.34) | 0.65 |  | 0.009 (-0.02, 0.04) | 0.55 |
| Subscapular Skinfold Thickness (mm) | 0.30 (-0.10, 0.69) |  | 0.14 |  | 0.13 (-0.09, 0.35) | 0.24 |  | 0.02 (-0.009, 0.04) | 0.22 |
| Suprailiac Skinfold Thickness (mm) | 0.11 (-0.20, 0.42) |  | 0.47 |  | 0.06 (-0.11, 0.22) | 0.51 |  | 0.006 (-0.01, 0.03) | 0.49 |
| Midthigh Skinfold Thickness (mm) | 1.43 (0.47, 2.4) |  | **0.004** |  | 0.53 (-0.004, 1.06) | 0.05 |  | 0.06 (0.004, 0.12) | **0.04** |

**Supplemental Table 5.** Beta coefficients and 95% confidence intervals (CIs) from multivariable linear regression analysis used to examine the associations between measure of infant growth with measures of alpha-diversity. Models adjusted for birth weight, birth length, and infant antibiotic exposure during the first month of life. *P*-values in bold denote statistical significance for *p*-values <0.05.

**Supplemental Table 6. Greater Gut Bacterial Alpha-Diversity were Associated with Rapid Infant Growth from Birth to 12-Months of Age after Adjusting for Infant Diet**

|  |  | **Adjusted OR**  **(95% CI)** | ***p*-value** |
| --- | --- | --- | --- |
| ***Alpha-Diversity Indices*** |  |  |  |
| Shannon diversity |  | 1.59 (0.90, 2.91) | 0.12 |
| Faith’s PD |  | 1.32 (0.97, 1.83) | 0.09 |
| Richness |  | 1.03 (1.00, 1.07) | 0.06 |

**Supplemental Table 6.** Multivariable odds ratios (OR) and 95% confidence intervals (CI) were calculated for these alpha-diversity indices, based on logistic regression with non-rapid growers as the referent, adjusting for birth length, birth weight, and infant diet (breastfeeding per day and formula feedings per day) during the first month of life. *P*-values in bold denote statistical significance for p<0.05.

**Supplemental Table 7**. **Infant Gut Bacterial Alpha-Diversity and Measures of Infant Growth after Adjusting for Infant Diet**

|  | **Shannon Diversity** | | |  | **Faith’s PD** | |  | **Richness** | |
| --- | --- | --- | --- | --- | --- | --- | --- | --- | --- |
|  | **Beta**  **(95% CI)** |  | ***p*-value** |  | **Beta**  **(95% CI)** | ***p*-value** |  | **Beta**  **(95% CI)** | ***p*-value** |
| ***Growth Measures***  ***(Birth to 12-Months)*** |  |  |  |  |  |  |  |  |  |
| Weight-for-Age z-score | 0.19 (-0.06, 0.44) |  | 0.14 |  | 0.12 (-0.01, 0.26) | 0.07 |  | 0.01 (-0.002, 0.03) | 0.09 |
| Weight-for-Length z-score | 0.17 (-0.06, 0.40) |  | 0.14 |  | 0.12 (-0.003, 0.24) | 0.06 |  | 0.014 (-0.00003, 0.03) | 0.51 |
| BMI z-score | 0.15 (-0.09, 0.39) |  | 0.21 |  | 0.12 (-0.005, 0.25) | 0.06 |  | 0.01 (0.0003, 0.03) | **0.046** |
| ***Anthropometrics***  ***(12-Months)*** |  |  |  |  |  |  |  |  |  |
| Weight (kg) | 0.22 (-0.01, 0.45) |  | 0.06 |  | 0.10 (-0.02, 0.23) | 0.11 |  | 0.01 (-0.002, 0.025) | 0.10 |
| Length (cm) | 0.25 (-0.28, 0.78) |  | 0.36 |  | 0.04 (-0.24, 0.33) | 0.76 |  | 0.002 (-0.03, 0.03) | 0.90 |
| Tricep Skinfold Thickness (mm) | -0.29 (-0.81, 0.24) |  | 0.28 |  | 0.02 (-0.26, 0.31) | 0.88 |  | 0.005 (-0.03, 0.04) | 0.73 |
| Subscapular Skinfold Thickness (mm) | 0.28 (-0.14, 0.70) |  | 0.19 |  | 0.15 (-0.08, 0.38) | 0.19 |  | 0.02 (-0.008, 0.04) | 0.17 |
| Suprailiac Skinfold Thickness (mm) | 0.0008 (-0.32, 0.32) |  | 1.00 |  | 0.04 (-0.13, 0.21) | 0.64 |  | 0.005 (-0.01, 0.02) | 0.59 |
| Midthigh Skinfold Thickness (mm) | 0.85 (-0.13, 1.83) |  | 0.09 |  | 0.34 (-0.19, 0.88) | 0.20 |  | 0.045 (-0.01, 0.10) | 0.13 |

**Supplemental Table 7.** Beta coefficients and 95% confidence intervals (CIs) from multivariable linear regression analysis used to examine the associations between measure of infant growth with measures of alpha-diversity. Models adjusted for birth weight, birth length, and infant diet (breastfeeding per day and formula feedings per day) during the first month of life. *P*-values in bold denote statistical significance for *p*-values <0.05.

|  | **Differentially Abundant**  **Gut Microbes*** | | |  |
| --- | --- | --- | --- | --- |
|  | **Beta**  **(95% CI)** |  | ***p*-value** |  |
| ***Growth Measures***  ***(Birth to 12-Months)*** |  |  |  |  |
| Weight-for-Age z-score | 0.093 (0.021, 0.17) |  | **0.012** |  |
| Weight-for-Length z-score | 0.05 (-0.042, 0.14) |  | 0.28 |  |
| BMI z-score | 0.066 (-0.014, 0.15) |  | 0.11 |  |
| ***Anthropometrics***  ***(12-Months)*** |  |  |  |  |
| Weight (kg) | 0.12 (0.059, 0.18) |  | **0.0001** |  |
| Length (cm) | 0.038 (-0.10, 0.18) |  | 0.59 |  |
| Tricep Skinfold Thickness (mm) | 0.12 (-0.010, 0.26) |  | 0.07 |  |
| Subscapular Skinfold Thickness (mm) | 0.090 (-0.014, 0.20) |  | 0.089 |  |
| Suprailiac Skinfold Thickness (mm) | 0.15 (0.073, 0.23) |  | **0.0002** |  |
| Midthigh Skinfold Thickness (mm) | 0.38 (0.12, 0.63) |  | **0.004** |  |

**Supplemental Table 8. The Newborn Gut Microbiota was Associated with Measures of Infant Growth after Adjusting for Infant Diet**

**Supplemental Table 8.** Beta coefficients and 95% confidence intervals (CIs) from multivariable linear regression analysis used to examine the associations between measure of infant growth with gut microbial composition at 1-month of age. *Differentially abundant gut microbes are defined as a curated selection of the log-ratio of the top- and bottom 40% ranked sOTUs that were differentially associated with rapid infant growth in the first year of life. P-values in bold denote statistical significance for those <0.05

|  | **Differentially Abundant**  **Gut Microbes*** | | |  |
| --- | --- | --- | --- | --- |
|  | **Beta**  **(95% CI)** |  | ***p*-value** |  |
| ***Growth Measures***  ***(Birth to 12-Months)*** |  |  |  |  |
| Weight-for-Age z-score | 0.010 (-0.08, 0.10) |  | 0.83 |  |
| Weight-for-Length z-score | 0.047 (-0.055, 0.15) |  | 0.36 |  |
| BMI z-score | 0.045 (-0.042, 0.13) |  | 0.31 |  |
| ***Anthropometrics***  ***(12-Months)*** |  |  |  |  |
| Weight (kg) | 0.041 (-0.032, 0.11) |  | 0.26 |  |
| Length (cm) | -0.014 (-0.19, 0.16) |  | 0.88 |  |
| Tricep Skinfold Thickness (mm) | 0.082 (-0.079, 0.24) |  | 0.31 |  |
| Subscapular Skinfold Thickness (mm) | 0.0029 (-0.12, 0.13) |  | 0.96 |  |
| Suprailiac Skinfold Thickness (mm) | 0.0004 (-0.090, 0.091) |  | 0.99 |  |
| Midthigh Skinfold Thickness (mm) | 0.12 (-0.19, 0.43) |  | 0.46 |  |

**Supplemental Table 9. The 6-month Gut Microbiota *and* Measures of Infant Growth**

**Supplemental Table 9.** Beta coefficients and 95% confidence intervals (CIs) from multivariable linear regression analysis used to examine the associations between measure of infant growth with gut microbial composition at 6 months of age. *Differentially abundant gut microbes are defined the log-ratio of features associated with rapid- and non-rapid growth at 1-month in the 6-month gut microbiota samples. P-values in bold denote statistical significance for those <0.05.

**Supplemental Table 10. Characteristics of Mother-Infant Dyads from the Southern California Mother’s Milk Study, Subset of Infants with Sequencing Data Available for 6-Months of Age, at 1-Month of Infant Age**

|  | **Overall Sample**  **at 1-Month**  **(n = 132)** | **Participants with**  **Sequencing Data at 6-months**  **(n = 89)** | ***p*-value** |
| --- | --- | --- | --- |
| ***Maternal Characteristics*** |  |  |  |
| Age at Infant Birth (years) | 29.5 ± 6.3 | 30.15 ± 6.47 | 0.47 |
| Pre-Pregnancy BMI (kg/m^2^) | 28.4 ± 5.7 | 28.36 ± 5.65 | 0.99 |
| Delivery (vaginal^a^, caesarian, %vaginal) | 98, 34, 74.2% | 70, 19, 78.7% | 0.15 |
| SES Index^b^ | 26.9 ± 12.2 | 27.64 ± 11.83 | 0.65 |
| Pre-Pregnancy Weight Status |  |  |  |
| Normal Weight | 37, 28.0% | 23, 25.8% | 0.62 |
| Overweight | 50, 37.9% | 36, 40.4% |  |
| Obese | 45, 34.1% | 30, 33.7% |  |
| Weight Status at 1-Month Postpartum |  |  |  |
| Normal Weight | 23, 17.4% | 14, 15.7% | 0.70 |
| Overweight | 48, 36.4% | 34, 38.2% |  |
| Obese | 61, 46.2% | 41, 46.1% |  |
| ***Maternal Dietary Measures at 1-Month*** |  |  |  |
| Total energy intake (kcals) | 1724.9 ± 534.73 | 1676.75 ± 521.73 | 0.51 |
| Fat intake (g) | 58.55 ± 23.09 | 56.46 ± 22.05 | 0.50 |
| Protein intake (g) | 76.487 ± 23.305 | 76.10 ± 24.65 | 0.91 |
| Carbohydrate intake (g) | 229.5 ± 81.701 | 222.85 ± 78.19 | 0.54 |
| ***Infant Characteristics*** |  |  |  |
| Age (days) | 32.8 ± 5.2 | 33.30 ± 5.78 | 0.53 |
| Infant Sex (female^a^, male, %female) | 72, 60, 54.5% | 49, 40, 55.1% | 1.00 |
| Delivery |  |  |  |
| On-Time (n, %)^a^ | 64, 48.5% | 43, 48.3% | 0.98 |
| Late (n, %) | 36, 27.3% | 24, 27.0% |  |
| Early (n, %) | 32, 24.2% | 22, 24.7% |  |
| Birth Weight (kg) | 3.4 ± 0.42 | 3.39 ± 0.40 | 0.90 |
| Birth Length (cm) | 50.5 ± 2.4 | 50.48 ± 2.40 | 0.90 |
| Weight (kg) | 4.6 ± 0.49 | 4.63 ± 0.47 | 0.84 |
| Length (cm) | 54.2 ± 1.8 | 54.06 ± 1.88 | 0.68 |
| Weight for Length z-Score | 0.65 ± 1.3 | 0.75 ± 1.45 | 0.59 |
| Body Mass Index z-Score | 0.59 ± 1.1 | 0.65 ± 1.13 | 0.67 |
| Antibiotics at 1-month (no^a^/yes, %yes)^c^ | 116, 15, 88.6% | 81, 7, 7.87% | 0.13 |
| ***Infant Dietary Measures*** |  |  |  |
| Breast Feedings/Day (≥8^a^, <8, % ≥8) | 97, 35, 73.5% | 68, 21, 76.40% | 0.38 |
| Formula Feeding/Day (yes/no^a^) | 51, 81, 38.6% | 52, 37, 58.43% | 0.42 |
| Age of Solid Food Introduction (months) | 5.8 ± 1.5 | 5.56 ± 1.08 | 0.26 |

**Supplemental Table 10.** Baseline (1-month) characteristics the full sample of Hispanic mother-infant dyads from the Southern California Mother’s Milk Study (n = 132) and the subset of the sample (n = 89) which also had sequencing data available at 6-months of infant age. Pre-pregnancy BMI, infant sex, birth weight and birth length are also shown. Data are reported mean and standard deviation (SD) unless otherwise noted. Total samples sizes include ^b^n=130 and n=88 for the “Overall Sample at 1-Month” and “Participants with Sequencing Data at 6-months”, respectively. Total samples sizes also include ^c^n=131 and n=89 for the “Overall Sample at 1-Month” and “Participants with Sequencing Data at 6-months”, respectively. For continuous variables, independent parametric or non-parametric t-tests were used to test for differences between the overall sample and the 6-month subset. For categorical variables, chi-square tests were used to test for differences between the overall sample and the 6-month subset.
